# Trends and the Economic Effect of Asbestos Bans and Decline in Asbestos Consumption and Production Worldwide

**DOI:** 10.3390/ijerph15030531

**Published:** 2018-03-16

**Authors:** Lucy P. Allen, Jorge Baez, Mary Elizabeth C. Stern, Ken Takahashi, Frank George

**Affiliations:** 1 NERA Economic Consulting, 1166 Avenue of Americas, New York, NY 10036, USA; lucy.allen@nera.com; 2 NERA Economic Consulting, 200 S. Biscayne Boulevard, Suite 950, Miami, FL 33131, USA; jorge.baez@nera.com; 3 NERA Economic Consulting, 360 Hamilton Avenue, White Plains, NY 10601, USA; 4 Asbestos Diseases Research Institute, University of Sydney, Gate 3 Hospital Road, Concord NSW 2139, Australia; ken.takahashi@sydney.edu.au; 5 World Health Organization (WHO), Regional Office for Europe, European Centre for Environment and Health, Platz der Vereinten Nationen 1, 53113 Bonn, Germany; georgef@who.int

**Keywords:** asbestos, bans, gross domestic product, employment, mesothelioma

## Abstract

Although some countries have reduced asbestos consumption and instituted bans, other countries continue to produce and consume asbestos even as asbestos-related deaths mount and the associated societal costs are high. Asbestos production and consumption has declined globally; the number of bans has increased; and the speed at which countries have tapered off consumption has increased. Using country-level data, we study the economic impact of historical changes in the production and use of asbestos. We compare changes in gross domestic product (GDP) following the enactment of asbestos bans. We do not find any significant effect on GDP following an asbestos ban. In a regional case study, we compare changes in GDP and employment with changes in asbestos production. Regional-level data revealed a temporary employment decline at the local level that was then reversed.

## 1. Introduction

Asbestos has been used for centuries because it is inexpensive and durable. Historically, asbestos has had more than 3000 different applications, primarily in construction materials and in a wide range of friction products [1]. However, asbestos has been linked with the development of deadly diseases, such as mesothelioma, lung cancer, and asbestosis. The health effects of asbestos use have been well-studied, particularly at the individual level, demonstrating a link between incidence of mesothelioma and prior exposure to asbestos [2]. These health effects have also been observed at the country level. Mortality rates of asbestos-related disease in the early 2000s have been correlated to aggregate asbestos use during the 1960s—the more asbestos consumption per capita by a country in the 1960s, the greater the mortality from asbestos-related diseases in that country in the early 2000s [3]. In addition, there is increasing evidence that reductions in asbestos consumption at the country level (often as the result of bans) have been associated with a subsequent decrease of rates in asbestos-related diseases. Countries that imposed asbestos bans reduced their asbestos use more quickly than those without bans, and a change in asbestos use between 1970 and 1985 was a significant predictor of the change in annual mesothelioma mortality rates between 1996 and 2005 [4]. Moreover, the incidence of mesothelioma (when measured at the same age) is lower for groups entering the workforce after a ban than for those who entered prior to a ban [5].

Estimates of the annual global health care costs associated with the health effects of asbestos range from US$ 2.4–3.9 billion, excluding the additional costs of pain, suffering, and welfare losses [6,7,8]. In particular, these estimates do not include costs associated with non-cancerous asbestos-related diseases such as asbestosis, any adjustment for years of life or years of healthy life lost due to asbestos-related diseases, or the economic burden due to asbestos-related diseases [9]. WHO and ILO recommend that the most effective way to eliminate asbestos-related diseases is to ban the use of all forms of asbestos [10,11].

Countries started banning asbestos in the 1970s, with partial asbestos bans [12]. By the mid-1980s, individual countries began instituting full bans, and in 2005, the European Union completely banned asbestos use by all its 25 member states [13]. As of 2013, 67 countries had instituted either partial or complete bans on asbestos.

Although the health effects of asbestos use have been well studied, the economic effects of asbestos bans and decreased consumption/production have been only rarely studied. A few researchers have estimated the potential economic impact of proposed asbestos bans for an individual country [14,15], but little has been written on the actual economic impact after a ban has been imposed. Potential adverse impacts predicted following a ban include negative economic effects in the asbestos extraction industry [14] or competition in the construction industry from neighboring countries still manufacturing asbestos-containing products [15].

We therefore analyzed whether the historical bans had any negative economic impact on these countries [16], both at the national level across countries and at the regional level in a case study for one country.

## 2. Materials and Methods

To assess asbestos production and consumption by countries over time, we obtained data on annual asbestos production and consumption from 1950 to 2013 from the U.S. Geological Survey (USGS) [17]. The USGS data report production/consumption by country every 10 years through 1970, every 5 years between 1970 and 1995, and then every year starting in 1995. These same data have been relied on by other researchers studying historical asbestos consumption across countries [18]. We identified countries that consumed more than 250,000 tons historically and for those countries that have transitioned away from asbestos, we assessed the time it took to shift away from asbestos consumption, specifically quantifying the number of years from peak consumption to 25% of peak consumption, as well as assessing how the transition time has changed over time. To control for countries that are continuing to use asbestos and whose consumption either has not yet peaked or has not yet declined to 25% of peak consumption, we ran a Cox proportional hazard model, testing the likelihood consumption would drop to 25% of peak consumption in any given year, controlling for the year consumption peaked.

To identify the countries that have enacted a ban on asbestos, we obtained data from the International Ban Asbestos Secretariat, which provides a list of complete and partial bans by country for the 67 countries with any type of asbestos ban [12].

To quantify the impacts of changes in asbestos production and consumption on economic activity, we obtained data on Gross Domestic Product (GDP) and GDP per capita for the producing and consuming countries from the United Nations in 2005 U.S. dollars [19]. In particular, we conducted an econometric analysis applying a difference-in-differences approach, using countries with bans to assess whether these bans were associated with any impact on economic activity. Due to data constraints, the analysis was limited to the country-level. The model is specified

$$\Delta GDP_{i,t}=\alpha_{i}+\gamma_{t}+\beta_{1}\cdot ban_{i,t}+\beta_{2}\cdot X_{i,t}+\varepsilon_{i,t},$$

where

$\Delta GDP_{i,t}$ = GDP growth
$\alpha_{i}$ = Country fixed effect
$\gamma_{t}$ = Time fixed effect
$ban_{i,t}$ = A dummy variable that equals one for years when country i has an asbestos ban in place and is zero otherwise
$\beta_{1}$ = Differences-in-differences effect of the asbestos ban
$X_{i,t}$ = Control variable (e.g., asbestos production or consumption)
$\beta_{2}$ = Coefficient on control variable
$\varepsilon_{i,t}$ = Residual term

To perform a regional case study of the potential economic impact of decreased production, we obtained data on GDP, population, and employment from Statistics Canada at the country, province, and local level [20]. Asbestos production in Canada, which was primarily concentrated in the province of Quebec, peaked in 1970 at 1.5 million metric tons [17]. By 2000, production had declined to 300,000 metric tons and continued to decline until the last two mines were closed in 2011 [17,21]. In 2012, the provincial government announced plans to invest funds in “economic diversification of the asbestos mining region” [22]. To assess the regional impact of the mine closures, we compared the change in per capita GDP growth in Quebec around the closure of the mines to the change in per capita GDP growth in the rest of Canada, since most of Canada’s asbestos production was concentrated in one province, Quebec. Additionally, Canadian employment data were available at a more local level, for the two regions within Quebec in which the mines were located, Estrie and Chaudière-Appalaches. For these regions, we assessed changes in employment levels following the closure of the mines.

## 3. Results

**Figure 1:**
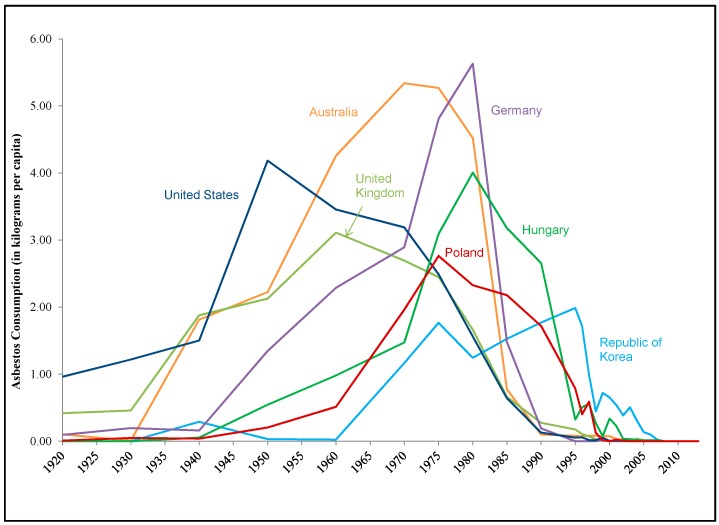
Per Capita Asbestos Consumption of Individual Countries, 1920–2013. Notes and Sources: Asbestos consumption data from the U.S. Geological Survey. Excludes negative asbestos consumption values.

**Figure 2:**
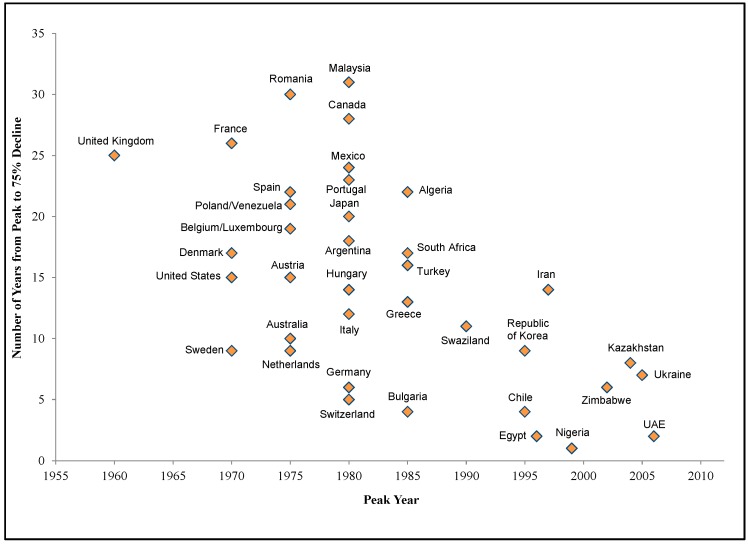
Time from Peak Consumption to 75% Decline. Notes and Sources: Asbestos consumption data from the U.S. Geological Survey. Excludes countries that consumed less than or equal to 250,000 metric tons in total between 1920 and 2013, and current consumers whose consumption has not declined below 25% of peak consumption.

Figure 1 demonstrates the individual consumption patterns for a sample of countries, in which consumption grows steadily, peaks, and then declines. Figure 2 shows the year in which asbestos consumption peaked for the 38 countries that were past users of asbestos, along with the number of years each country took to transition away from using asbestos (i.e., the number of years between peak consumption and when consumption had declined to 25% of the peak). For those countries that have transitioned away from asbestos, the time from peak consumption to 25% of peak consumption has been decreasing over time.

The observed negative trend in Figure 2 does not take into account the current consumers whose consumption either has not yet peaked or has not yet declined below 25% of peak consumption. Including the current consumers as well as the countries that have already transitioned away from asbestos, we found that the likelihood that consumption drops to 25% of peak consumption in any given year increases by 4.1% for every later year that a country’s consumption peaks, and the increased likelihood of a decline in consumption was statistically significant. Table 1 below displays the results from a Cox proportional hazard model that relates the duration of time for a country to transition from peak to 25% below peak to a country’s peak year. We found a statistically significant effect (*p* = 0.025). We statistically tested the proportional hazard assumption that underlies this model and found that that we cannot reject the null hypothesis that the assumption does not hold (*p* = 0.196).

Table 2 contains the results from our differences-in-differences model that relates GDP growth to the implementation of asbestos bans. These models indicate that there is not a statistically significant impact on GDP growth of asbestos bans. Column 1 of Table 2 presents the main differences-in-differences model, using as a sample, countries that consume asbestos in our dataset. The model includes country and year fixed-effects, as well as the dummy variable which indicates whether an asbestos ban is currently in place in country i at time t. Table 2 also includes two additional specifications, one which includes a control for asbestos consumption in country i at time t (Column 2) and another which includes a control for asbestos production in country i at time t (Column 3). Overall, we found that there was no significant effect of the bans on GDP growth.

**Figure 3:**
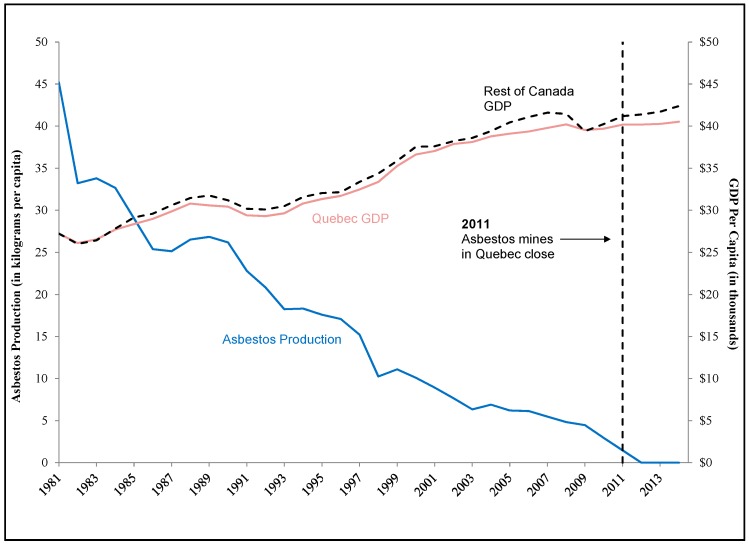
Quebec Asbestos Production and GDP Per Capita, 1981–2014. Notes and Sources: Asbestos production data from the U.S. Geological Survey. GDP and population data from Statistics Canada. GDP data are in constant 2007 prices. Rest of Canada GDP per capita excludes Quebec’s GDP and population and is pegged to the value of Quebec’s GDP per capita in 1981. The majority of Canada’s asbestos production took place in Quebec.

**Figure 4:**
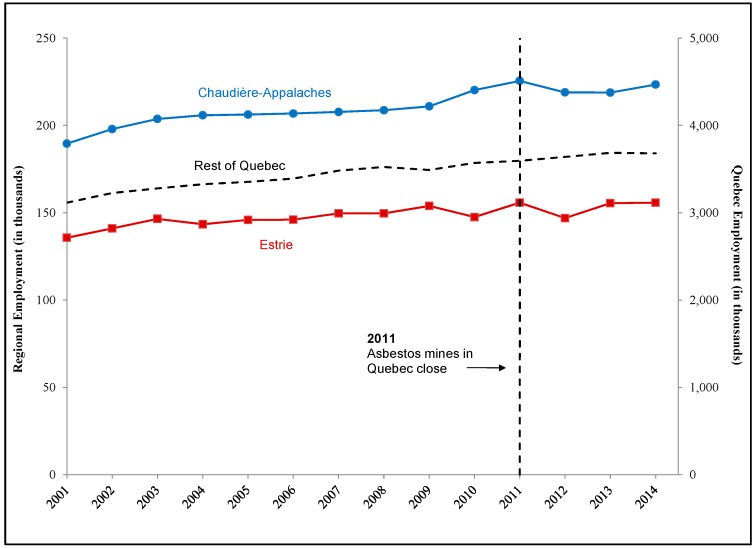
Employment in Chaudère-Appalaches and Estrie, Quebec. Notes and Sources: Employment data from Statistics Canada.

Beyond this cross-country analysis, we also considered a regional case study. In this analysis, we also did not observe a negative impact on GDP in the province of Quebec following closure of the mines (see Figure 3). However, following closure of the mines, total employment in each region where the mines were located dropped—from 156,000 people employed to 147,000 people employed in the Estrie region (a decline in the employment rate (number of people employed per 100 members of the population) from 59.5% to 55.6%) and from 226,000 people employed to about 219,000 people in the Chaudière-Appalaches region (a decline in the employment rate from 65.6% to 63.4%) [20]. Similar declines in employment did not occur in the rest of Quebec. However, within two years, the employment levels in both regions had returned to pre-closure levels (see Figure 4).

## 4. Discussion

The international market for asbestos has been shrinking, both in terms of the quantity sold and the number of countries participating. Annual asbestos production and consumption worldwide have been in decline since their peak in 1980, at approximately 4.8 million metric tons. By 2000, annual asbestos production and consumption had fallen to approximately 2.0 million metric tons, where it has remained for the past decade [17]. Over this same period, the number of countries producing and consuming asbestos became more concentrated [17]. In 1980, 20 countries produced asbestos and 90 countries consumed the mineral. By 2013, asbestos producers were down to six countries (Russian Federation, China, Brazil, Kazakhstan, India and Argentina), with the first four accounting for 99 percent of annual production; asbestos consumers were down to 25 countries, with 10 accounting for 90 percent of annual consumption (China, the Russian Federation, India, Brazil, Thailand, Kazakhstan, Indonesia, Vietnam, Uzbekistan, and Turkmenistan) and the first three alone accounting for over 60 percent of the annual consumption.

An examination of consumption patterns for individual countries over time shows a similar pattern across countries that no longer consume asbestos: a steady growth until a peak is reached and then a decline. This pattern has occurred at different times for different countries: asbestos consumption peaked in the 1960s in the United Kingdom, in the 1980s in Hungary and Germany, and in the mid-1990s in the Republic of Korea.

The time countries take to transition away from asbestos appears to be shortening in recent years. For example, the United Kingdom, which had its peak consumption in 1960, took 25 years to transition away from using asbestos. Hungary, which peaked in 1980, took 14 years to transition away from asbestos, while Chile, which peaked in 1995, took four years. Countries that have reached the peak of their asbestos consumption more recently are, on average, taking fewer years to transition away from asbestos, even accounting for the current consumers. The later their peak in use, the more quickly countries are transitioning away from asbestos. The increasing speed with which countries have shifted away from asbestos use suggests that the pace of contraction in the industry may also increase.

For the countries that have banned asbestos, although we did not observe a statistically significantly impact on their GDP growth as a result of the bans, the power of this country-level analysis to detect effects is limited in part due to the fact that asbestos production and/or consumption were typically not a substantial factor in country-level GDP. For example, the mining and utilities sector (of which asbestos was one aspect) represented only 3% of GDP in Italy in the years prior to its ban. As a result, country-level statistics may well hide even large impacts on specialized economies or communities. Yet, to the extent that asbestos may similarly not be a large part of the economies of the current asbestos consumers, these results may still offer insight into the likely impact on their economies, if the current consumers also elected to ban asbestos.

At a regional level, while the end of asbestos production did not have an impact on provincial GDP and the employment impacts were temporary, we did not study the drivers behind the subsequent increased employment in these regions post-closure of the mines. Data are limited at the local level, making it difficult to observe and control for all factors that may influence a ban’s effect on a particular community. Therefore, whether population changes/migration or government intervention may have played a role in each Canadian region’s recovery is not known. da Silva hypothesized that potential negative impacts might be mitigated through public policies in the affected region, including the development of other industries, such as the growth of the non-asbestos, fiber-cement industry or the asbestos-removal industry [14]. To the extent that government activities may have helped mitigate the employment declines, an understanding of such strategies (if implemented) would be helpful for other countries considering banning asbestos. As a result, more studies may be needed to identify and quantify costs that may be observable only at a more local level. Such studies might help countries identify and target regional policies to address any short-term local impacts from the declines in consumption and production.

Countries that are still producing and using asbestos today may experience other costs in addition to substantial health costs, such as remediation and removal costs, as well as compensation costs, which may include significant litigation costs for some countries. The extent to which compensation for asbestos-related diseases occurs through litigation is likely to depend on the litigation environment within each country [23]. Data on these costs are not readily available for countries currently using asbestos. Based on a comparison of worldwide asbestos consumption from the early 1900s to the present, however, the total asbestos consumption of current consumers has surpassed the amount of asbestos consumed by the United States of America, a country for which data on historical costs are available [17]. Current consumers have already used over twice as much asbestos as the United States in its many decades of past use. Studies have estimated the costs of the United States’ past use of asbestos as billions of dollars annually. In particular, the annual medical costs from mesothelioma in the United States have been estimated at US$ 1.9 billion, and annual remediation costs have been estimated at approximately US$ 3.0 billion: almost US$ 5.0 billion combined, before any estimate of associated productivity losses [7,24]. Asbestos litigation costs in the United States, which are a proxy for compensation-related costs, have been estimated at another US$ 2.3 billion per year [25]. Although the experience of past asbestos consumers such as the United States may be on the higher end of the scale, a modest extrapolation will still lead to the assumption that continued consumption and production of asbestos is likely to lead to substantial medical and remediation costs, including removal and waste-management costs, as well as potential litigation and compensation costs. Indirect economic costs, such as loss of labor-force participation and reduced tax revenues present additional costs not reflected in these cost estimations.

## 5. Conclusions

The international market for asbestos is shrinking and, even accounting for current consumers, the speed at which countries have tapered off consumption has increased. As countries have shifted away from asbestos, we did not find an observable negative economic impact following the institution of bans using country-level data. To the extent that asbestos represents a similarly small share in the economies of the current consumers, a similar ban would not be expected to have a large economic impact at the national level. Where relevant regional-level data were available, we did not observe a persistent effect at a local level following declines in asbestos consumption or production. Whereas the shift away from asbestos has not had an observable persistent negative economic impact, continued use of asbestos is expected to result in substantial costs, including health costs as well as remediation/removal costs and potential litigation costs.

## Figures and Tables

**Table 1:** Results of Cox proportional hazards model

Number of countries in sample			49	
Number of countries that have transitioned away from asbestos			38	
**Variable**	**Coefficient**	**Std. Error**	***p*-Value**	**95% CI**
Peak year	0.041	0.018	0.025	0.005–0.076

Notes and Sources: Asbestos consumption data from the U.S. Geological Survey. Excludes countries that consumed less than or equal to 250,000 metric tons in total between 1920 and 2013.

**Table 2:** Differences-in-differences model results

Model	1	2	3
**Estimated Coefficients (*p*-Values)**			
Diff-in-diff estimator	−0.012 (0.302)	−0.015 (0.55)	0.009 (0.866)
Asbestos control		0.0008 (0.004)	−0.002 (0.864)
Fixed effects	Year, Country	Year, Country	Year, Country
R-squared	0.35	0.43	0.39
Country sample	Consumer	Consumer	Producer

Notes and Sources: Asbestos consumption and production data obtained from US. Geological Survey. Data on asbestos bans obtained from The International Ban Asbestos Secretariat.
